# Investigation of Biotransformation Products of *p*-Methoxymethylamphetamine and Dihydromephedrone in Wastewater by High-Resolution Mass Spectrometry

**DOI:** 10.3390/metabo11020066

**Published:** 2021-01-25

**Authors:** Juliet Kinyua, Aikaterini K. Psoma, Nikolaos I. Rousis, Maria-Christina Nika, Adrian Covaci, Alexander L. N. van Nuijs, Νikolaos S. Τhomaidis

**Affiliations:** 1 Toxicological Center, Department of Pharmaceutical Sciences, Campus Drie Eiken, University of Antwerp, Universiteitsplein 1, 2610 Antwerp, Belgium; juliet.kinyua@outlook.com (J.K.); adrian.covaci@uantwerpen.be (A.C.); alexander.vannuijs@uantwerpen.be (A.L.N.v.N.); 2 Laboratory of Analytical Chemistry, Department of Chemistry, National and Kapodistrian University of Athens, Panepistimiopolis Zografou, 15771 Athens, Greece; katpsoma@chem.uoa.gr (A.K.P.); nrousis@chem.uoa.gr (N.I.R.); nika_mar-chr@chem.uoa.gr (M.-C.N.)

**Keywords:** wastewater-based epidemiology, new psychoactive substances, mephedrone, transformation products, liquid chromatography tandem mass spectrometry, target and suspect screening, retention time prediction model, activated sludge

## Abstract

There is a paucity of information on biotransformation and stability of new psychoactive substances (NPS) in wastewater. Moreover, the fate of NPS and their transformation products (TPs) in wastewater treatment plants is not well understood. In this study, batch reactors seeded with activated sludge were set up to evaluate biotic, abiotic, and sorption losses of *p*-methoxymethylamphetamine (PMMA) and dihydromephedrone (DHM) and identify TPs formed during these processes. Detection and identification of all compounds was performed with target and suspect screening approaches using liquid chromatography quadrupole-time-of-flight mass spectrometry. Influent and effluent 24 h composite wastewater samples were collected from Athens from 2014 to 2020. High elimination rates were found for PMMA (80%) and DHM (97%) after a seven-day experiment and degradation appeared to be related to biological activity in the active bioreactor. Ten TPs were identified and the main reactions were *O*- and *N*-demethylation, oxidation, and hydroxylation. Some TPs were reported for the first time and some were confirmed by reference standards. Identification of some TPs was enhanced by the use of an in-house retention time prediction model. Mephedrone and some of its previously reported human metabolites were formed from DHM incubation. Retrospective analysis showed that PMMA was the most frequently detected compound.

## 1. Introduction

In the last two decades, an increased production and use of new psychoactive substances (NPS) was observed globally. NPS are chemically modified compounds of conventional illicit drugs (i.e., cocaine, ecstasy, and amphetamine) producing similar effects. Cathinones, cannabinoids, benzodiazepines, and phenethylamines were of the main classes found in the market and hundreds of these substances were reported the last 15 years [1,2]. The synthetic phenethylamine drug, *p*-methoxymethylamphetamine (PMMA, C_11_H_17_NO), is structurally and pharmacologically related to 4-methoxyamphetamine (PMA) and 3,4-methylenedioxymethamphetamine (MDMA). It is found in tablets and capsules sold on the illicit market as a substitute for “ecstasy”, presenting nevertheless a slower onset of action and higher toxicity than MDMA [3,4]. Dihydromephedrone (DHM, C_11_H_17_NO) is identified both as metabolite and transformation product of mephedrone in several studies [5]. Mephedrone is a synthetic, psychoactive drug structurally derived from cathinone and it has appeared in drug markets since 2007. It supplemented and/or replaced MDMA, with its consumption associated with parties and night-clubs [6]. Both PMMA [7,8,9,10,11,12,13,14] and mephedrone [15,16,17,18] have been associated with fatal and non-fatal intoxications worldwide and their use was banned in many countries.

Many different sources are used by the European Monitoring Centre for Drugs and Drug Addiction (EMCDDA) to track NPS use and diffusion including population surveys, drug seizures, police intelligence, internet, forensic toxicology reports, and the wastewater-based epidemiology (WBE) approach [19]. WBE is used as a complementary tool to monitor spatial and temporal trends of illicit drug consumption in communities by EMCDDA [20]. Only a few studies investigated the occurrence of NPS in wastewater (i.e., [21,22,23,24,25,26]), but WBE is a rapidly evolving discipline and more research is expected in the near future. Monitoring NPS in wastewater is a challenging task, since [5,19,27]: (i) a large number of new compounds are produced in a limited time and new analytical methods are needed for these substances; (ii) the concentration levels and abundance in wastewater are expected to be low, because the consumption rates are low due to little popularity in the market (sensitive and selective methods are required); (iii) limited data are known with respect to NPS metabolism and excretion and, thus, selection of suitable WBE biomarkers is considered difficult; and (iv) biotransformation and stability in wastewater are not well understood.

The biotransformation and stability of NPS in wastewater has received little attention so far and most of the WBE studies have focused on in-sample stability [25,28]. Furthermore, the fate of NPS and their transformation products (TPs) in wastewater treatment processes is not thoroughly investigated yet, compared to other chemicals of emerging concern (i.e., pharmaceuticals, household chemicals, personal care products, and pesticides [29,30,31,32]). Metabolism and biotransformation studies of NPS are of high importance and should be promoted, since there is a need to choose the most representative compounds for WBE and to detect potential overlap of other biotransformation (i.e., microbial and mammalian) routes [19,33,34].

The increasing number of emerging NPS in recent years, the absence of reference standards, and/or their prohibitive cost has made high-resolution mass spectrometry (HRMS) instruments necessary in forensic, chemical (analytical and environmental), and clinical screening procedures [35]. HRMS systems and appropriate analytical strategies are required for the detection and identification of (new) TPs in wastewater [36]. Moreover, the use of retention time prediction models could be a complementary and very advantageous tool for the identification of suspect and unknown compounds [37]. However, it has been emphasized that prediction models cannot always distinguish isomers and isobaric compounds and, thus, the use of analytical standards are necessary for the confirmation of a suspect isomeric and isobaric compound in real samples [38].

The main objectives of this work were: (i) to perform transformation experiments with activated sludge and wastewater and to explore the formation of TPs from PMMA and DHM, applying target and suspect screening based on liquid chromatography quadrupole-time-of-flight mass spectrometry (LC-QToF-MS); (ii) to elucidate the structures of the candidate TPs, based on accurate mass and isotopic pattern using LC-QToF-MS and tentative interpretation of MS/MS spectra; (iii) to support and enhance (if possible) the identification level of the TPs using an in-house retention time prediction model; and (iv) to propose potential WBE biomarkers for the studied NPS.

## 2. Results

### 2.1. PMMA and DHM Degradation

**Figure 1:**
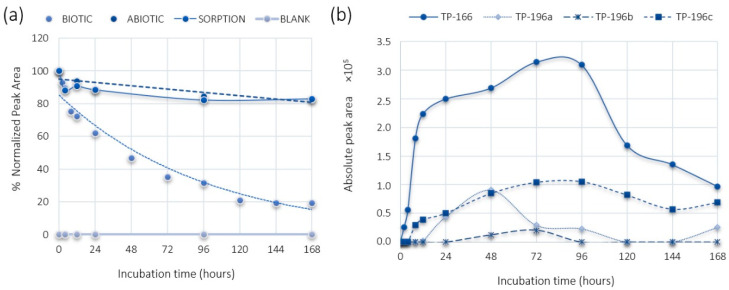
(**a**) PMMA degradation in different reactors; (**b**) PMMA TPs formation in the biotic reactor.

**Figure 2:**
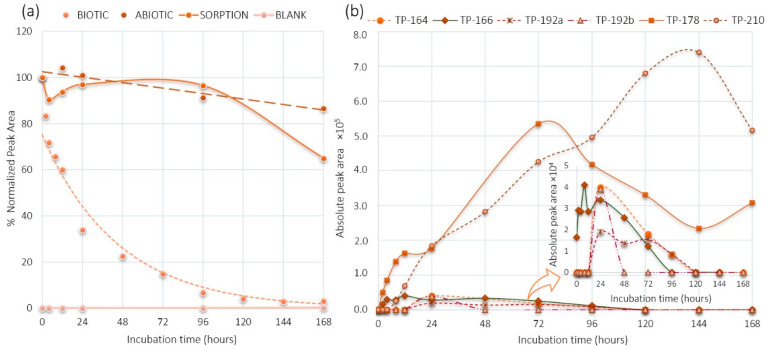
(**a**) DHM degradation in different reactors; (**b**) DHM TPs formation in the biotic reactor.

The aerobic degradation of PMMA in the activated sludge (biotic) reactor was deficient after a seven-day experiment (Figure 1). On the other hand, the aerobic degradation of DHM was almost 100% (Figure 2). The initial PMMA concentration (2 mg/L was the spiking level and 3 g/L the total suspended solids concentration) was decreased 50% during the first two and a half days and then decreased further reaching the maximum degradation rate of approximately 80% after five days, where it remained stable until the end of the experiment. DHM presented a faster degradation rate than PMMA, with a half-life of one day and an overall degradation up to 100% from the fourth day of the experiment. An exponential decrease was observed during the seven-day experiment.

The control reactors running in parallel showed limited losses. PMMA presented 18% losses in both the sorption and abiotic reactors and DHM 14% in the abiotic reactor after 168 h. The control experiments with diluted autoclaved sludge (sorption reactor) showed significant losses (45% after seven days). However, until the fourth day of the experiment, where DHM was completely degraded, negligible losses were observed (4%). Thus, the degradation of both PMMA and DHM can be related to biological activity in the active bioreactor.

The parameters pH and temperature remained constant throughout the experiment.

### 2.2. PMMA Biotransformation Products

Four TPs were found in the biotic reactor experiments, namely TP-166 and TP-196(a–c) (Table 1).

**Figure 3:**
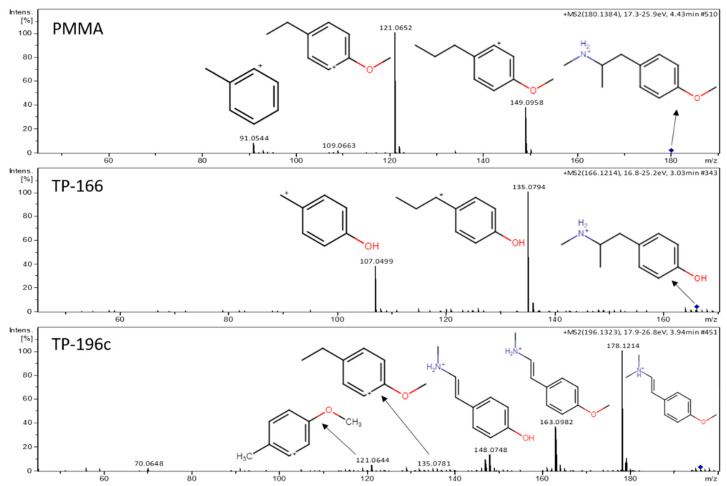
MS/MS spectra and fragmentation pattern of PMMA and two of its TPs.

The chromatographic peak of the TP-166 (*m/z* 166.1215) at 3.01 min could be attributed to two different compounds, the *N*-demethylation or *O*-demethylation product of PMMA according to the suspect database. This TP was observed after 2 h at relatively low levels (absolute peak area) (Figure 1) and according to accurate mass, retention time, and MS/MS spectra the TP-166 was the *O*-demethylation product of PMMA (Figure 3). The retention time prediction model calculated a t_R_ of 5.87 min for the *N*-demethylation product and 3.14 min for the *O*-demethylation one. Hence, the *N*-demethylation product was excluded as a potential TP according to the prediction model. Furthermore, the product ions of the MS/MS experiments corresponded to loss of NH_2_CH_3_ (*m/z* 135.0797) and a further loss of C_2_H_4_ (*m/z* 107.0491) (Figure 3), which matched the product ions of *p*-OH-methamphetamine [5,40].

Three peaks were observed for the TP-196(a–c) at 3.0, 3.5, and 3.9 min. TP-196a, TP-196b, and TP-196c were observed after 24 h, 48 h, and 8 h, respectively (Figure 1). Three possible structures were proposed, including two products of aliphatic hydroxylation reactions and one due to *N*-hydroxylation. Retention time prediction analysis calculated a range of 3.2–4.7 min as elution time for the three compounds and, thus, it was not possible to discern the isomeric forms of TP-196 by this model. The TP-196c was identified as a hydroxylation product of PMMA based on accurate mass and MS/MS spectra. The product ions for TP-196c resulted from the loss of a hydroxyl and a methyl group (*m/z* 178.1216), a further loss of the (N) methyl group (*m/z* 163.0981) and, finally, an additional (O) methyl loss (*m/z* 148.0736). The diagnostic product ions with *m/z* 121.0654, and *m/z* 135.0774 matched the alkyl benzene moieties with an intact methoxy group (Figure 3). These product ions were not distinctive to any of the three isomeric forms and the obtained fragmentation did not depend on the position of the hydroxyl substituent. Therefore, an identification level 3 was assigned, since the position of the hydroxyl group could not be confirmed. To the best of our knowledge, TP-196 has not been previously identified in relation to PMMA degradation.

### 2.3. DHM Biotransformation Products

Six TPs were found in the biotic reactor (Table 2). TP-210 and TP-178 were characterized as the major TPs and TP-166, TP-164, TP-192a, and TP-192b, as the minor TPs according to their abundance (Figure 2).

**Figure 4:**
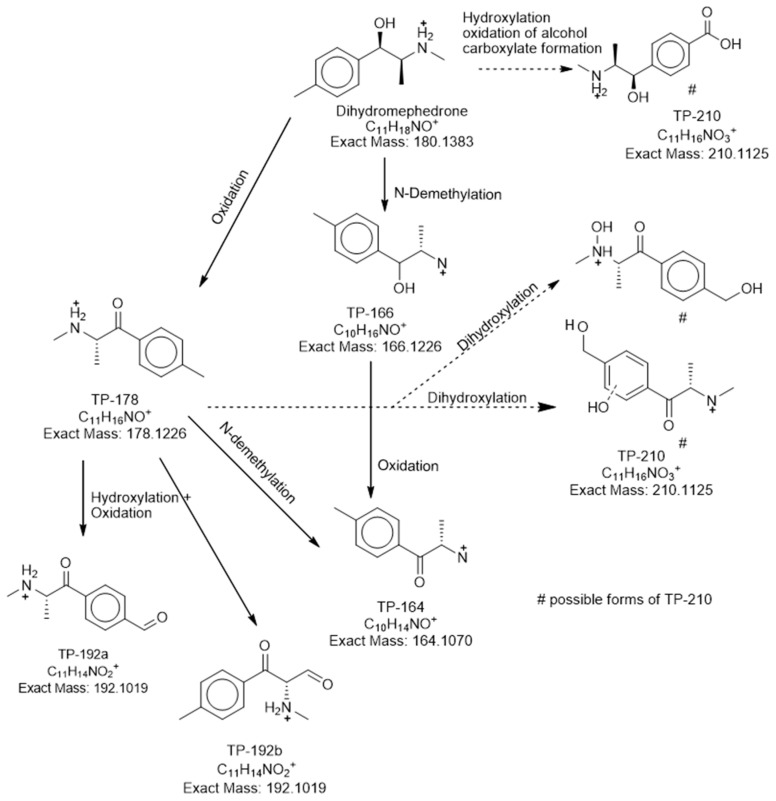
Proposed biotransformation pathway for DHM. Possible reactions are indicated by dotted arrows.

The TP-210 with *m/z* 210.113 and retention time of 1.82 min was detected after 8 h of incubation. Three isomeric compounds were proposed according to the suspect database, but the retention time prediction model was not able to distinguish the isomeric forms of TP-210. The predicted retention times (from 3.1 min to 3.3 min) of isomers were within the limits of the model [37] and, therefore, it could be assigned to one of the isomeric TPs. The formation of TP-210 (Figure 4) was proposed to be done by two biotransformation pathways. Oxidation of the primary alcohol on DHM was the first one, following the formation of a ketone group on the β-carbon (forming TP-178) and, after that, dihydroxylation. Two TP-210 structures could be formed: a hydroxylation on methylphenyl and primary amine groups or a hydroxylation on the benzene ring and methylphenyl group. The second probable biotransformation pathway for TP-210 could be done by three reactions. It is proposed a hydroxylation on the aliphatic methyl group, following by an oxidation of the alcohol to form an aldehyde group and, finally, a carboxylate formation (Figure 4). The last form matched 4-carboxy dihydromephedrone as it was described in human urinary experiments [15].

**Figure 5:**
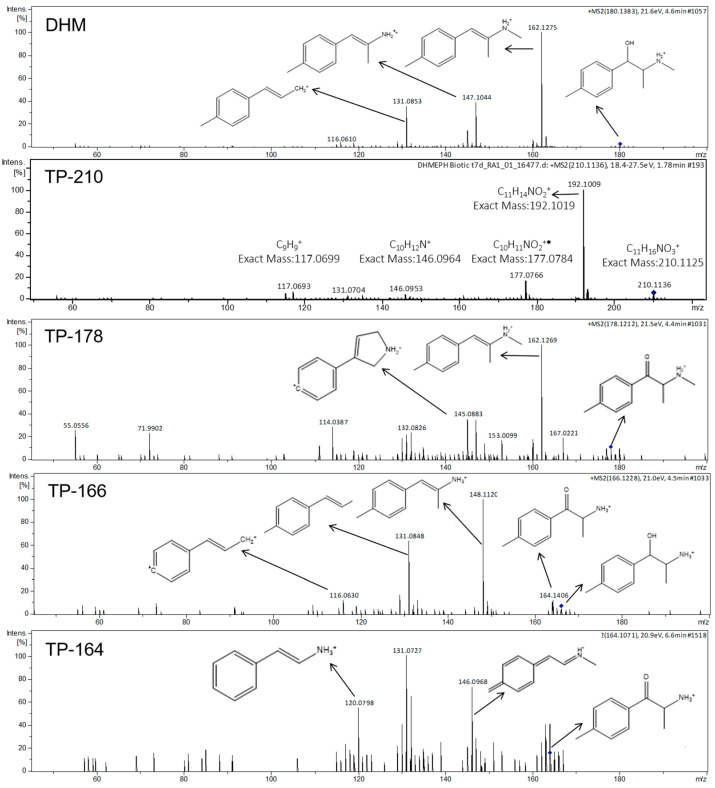
MS/MS spectra and fragmentation pattern of DHM, TP-210, TP-166, TP-178, and TP-164.

The TP-178 with *m/z* 178.1212 at 4.64 min was occurred after 2 h, getting its maximum point at 72 h and decreased gradually afterwards (Figure 2). TP-178 corresponded to the oxidation of the primary alcohol of DHM and taking into account the mass accuracy, retention time and MS/MS spectra, it was identified as mephedrone (Figure 5). Finally, an identification level of 1 was given, since it was confirmed with the corresponding reference standard.

TP-166 with *m/z* 166.1228 at 4.49 min was found at 2 h time-point, reaching its maximum peak at 12 h (Figure 2). TP-166 was characterized as the *N*-demethylated form (MS, MS/MS, retention time) of DHM (Figure 5) which was identified in rat and human studies as nor-dihydromephedrone [16,41].

TP-164 with *m/z* 164.1071 at 6.5 min was identified for the first time at 24 h and presented until the 96 h time-point (Figure 2). Two pathways were proposed for the formation of TP-164, the *N*-demethylation of TP-178 and/or the oxidation of TP-166 leading to the formation of a β-ketone group. Product ions of TP-164 with *m/z* 146.0968 (H_2_O loss) and *m/z* 131.0727 (CH_5_O loss) matched in vivo and in-sewer studies [5,15].

**Figure 6:**
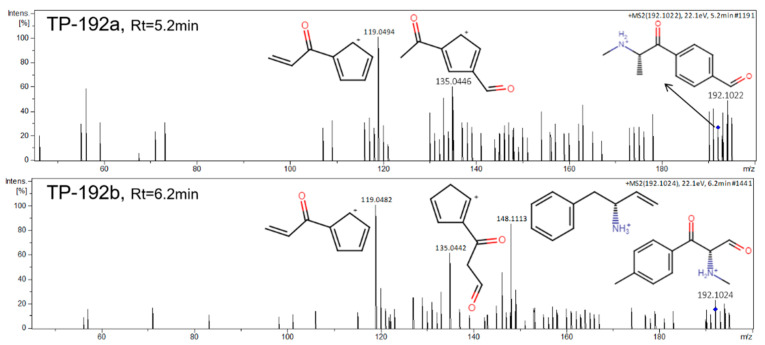
MS/MS spectra and fragmentation pattern of DHM TPs, TP-192a, and TP-192b.

TP-192a and TP-192b with *m/z* 192.1024 at 5.10 min and *m/z* 192.1022 at 6.15 min respectively were occurred at 24 h (Figure 2). MS/MS spectra of both TPs with their fragmentation can be found in Figure 6. Two different reaction pathways were proposed for the TP-192a and TP-192b (Figure 4). Firstly, formation of TP-192a involved the hydroxylation and then oxidation of the aromatic methyl group on TP-178 forming a ketone. Secondly, formation of TP-192b involved the hydroxylation and then oxidation on the aliphatic methyl of TP-178 forming a ketone. The proposed isomer TP-192a could be the hydroxytolylmephedrone with a further oxidation of the primary alcohol. TP-192 isomers have not been previously identified in mephedrone studies.

### 2.4. Occurrence of the Selected Compounds and Their TPs in Wastewater

Retrospective analysis of influent (IWW) and effluent wastewater (EWW) samples from Athens was executed for the detection of PMMA, DHM, and their TPs. Some results of PMMA have been published elsewhere [23,42]. PMMA was detected in wastewater from 2014 to 2020, except in the year 2019. PMMA TPs were never detected. DHM was only detected in both IWW and EWW in 2017. The DHM TPs, TP-178, TP-164, TP-192b, and TP-210 were not detected. The DHM TP-166 (nor-dihydromephedrone) was found in three EWW samples in 2020, and TP-192a in all EWW in 2015, 2016, and 2019.

## 3. Discussion

The degradation batch experiments with activated sludge presented high elimination rates for PMMA (80%) and DHM (~100%) and control reactors presented lower removal rates (20% for PMMA and 30% for DHM). Therefore, the degradation experiments were associated to a great extent to biological activity. Sorption and abiotic reactors for PMMA and the abiotic reactor for DHM showed small losses and this loss was due to sorption processes or reactions with sludge particles or other abiotic reactions (i.e., hydrolysis and volatilization). The control experiments with the sorption reactor showed important losses for DHM that can be attributed to sorption onto sludge particles most likely by a partial reactivation of the autoclaved sludge, since sludge can be contaminated during sampling [43].

During the biodegradation experiments eight TPs were formed. The main reactions for PMMA TPs were *O*-demethylation and hydroxylation, while oxidation, hydroxylation, and *N*-demethylation were observed for DHM TPs. One of the TPs formed (i.e., mephedrone) was confirmed with the corresponding reference standard. Furthermore, the PMMA TP-166 was confirmed as *p*-OH-methamphetamine, since its experimental spectrum matched with literature. For the additional TPs, probable structures based on diagnostic evidence were proposed. The retention time prediction model was used to assist identification (no reference standards were available) and was able to distinguish two isomeric compounds (*O*-demethylation product of PMMA).

Four PMMA TPs were found in the biotransformation experiments. The TP-166 corresponded to *p*-OH-methamphetamine which was confirmed as a human metabolite [40,44], and an in-sewer PMMA TP [5]. Additionally, it was found to be a methamphetamine metabolite [44]. These experiments suggested that *p*-OH-methamphetamine cannot be used as a WBE biomarker for PMMA. Additionally, it was formed early (2 h) at a relatively low concentration in the experiment, taking into account that a mean hydraulic residence time usually ranged from 1 to 12 h with a mean value of ~4 h [45]. Therefore, PMMA itself could be measured in untreated wastewater with the aim to assess human consumption. However, direct disposals should be considered when parent compounds are used as WBE biomarkers. To the best of our knowledge, the TP-196 has never been identified in the literature. An identification level of 3 was assigned and, thus, further research is needed to confirm its identity.

Six DHM TPs were detected and identified in the biotic reactor. TP-210, TP-164, and TP-166 were reported as human urinary metabolites [15,16] and TP-192a and TP-192b have never been reported previously in the literature. According to the fragmentation pathway of TP-210, the carboxylated form (4-carboxy dihydromephedrone) should be the most probable structure. However, an identification level 3 was assigned, since the position of the substituent hydroxyl group could not be confirmed (Figure 5). A reference standard is needed to confirm the above hypothesis. The TP-178 was confirmed as mephedrone, which was formed back from DHM incubation. Indeed, in-sewer experiments with the presence of biofilm reported mephedrone as highly unstable [5]. Mephedrone is currently used as a WBE biomarker [46,47,48], but its stability under different wastewater compositions could lead to different transformation rates [49]. This study illustrated how complicated a back-calculation model for mephedrone can be, since it was also formed from DHM incubation. Therefore, mephedrone consumption is possibly overestimated if the in-sewer dynamics and transformation processes are not taken into consideration. TP-164 could be identified as nor-mephedrone, since its characteristics matched in vitro, in vivo [15,16,41,50,51], and in-sewer [5] studies. However, it could not be confirmed without a commercial reference standard (Figure 5). Additionally, in vivo metabolism of mephedrone proposed the *N*-demethylation pathway as the main metabolic pathway [50] (Figure 4).

PMMA was the most frequently detected compound, presented in both IWW and EWW. Its occurrence in EWW showed that the WWTP was not able to eliminate it. A few studies have detected this compound in wastewater [23,26,42]. PMMA TP-166 was not detected, even if it was formed in a relatively short time (2 h). Its absence could be explained by the fact that it had low abundance during incubation experiments when the spiked PMMA concentration was high, compared to the usual levels found in wastewater. TP-196(a–c) were formed after 24 h, 48 h, and 8 h, respectively, thus, their presence in IWW was not expected, taking into account the mean hydraulic residence time. DHM was detected in all IWW and EWW samples in 2017. However, the parent compound mephedrone was not detected that year [23]. DHM TP-166 was detected in three EWW in 2020 and the signal to noise ratio was around four. The presence only in EWW could be due to its cleaner matrix compared to IWW, which significantly affects the method sensitivity, especially at such low concentration levels. TP-192a was detected only in EWW samples and it could be explained by its high formation time (24 h) in wastewater.

## 4. Materials and Methods

### 4.1. Chemicals and Reagents

Analytical standards of PMMA and DHM were purchased from LGC Standards SARL (Molsheim, France) and Cerilliant (Round Rock, TX, USA). Information on the reagents (methanol, acetonitrile, ethyl acetate, ammonium formate, ammonium acetate, formic acid, and distilled water) and sample preparation consumables can be found elsewhere [42].

### 4.2. Samples and Sampling Procedure

The samples (activated sludge, IWW and EWW) were collected from the wastewater treatment plant (WWTP) of Athens, Greece. Daily composite IWW and EWW were sampled during spring for eight or seven consecutive days from 2014 to 2020 and they were used for the retrospective analysis of PMMA, DHM, and their TPs. A population of 3,700,000 inhabitants is covered by the WWTP of Athens.

Samples were collected in high-density polyethylene bottles.

### 4.3. Biotransformation Batch Experiments

The individual biotransformation of PMMA and DHM was performed over seven-day batch experiments. The final concentration of PMMA and DHM to the bioreactors was 2 mg/L. More details about biotransformation batch experiments can be found to our previous publication [52].

### 4.4. Treatment of Wastewater Samples and Instrumental Analysis

Solid phase extraction was applied as pretreatment procedure and the analysis was done using LC-QToF-MS instrumentation. More details about the analytical methodology can be found elsewhere [42].

### 4.5. Identification of Transformation Products by Suspect Screening

Detection and identification of PMMA and DHM TPs was performed by a post-acquisition approach [36]. A suspect database of potential TPs was built using two in silico prediction tools, the Eawag-Biocatalysis/Biodegradation Database Pathway Prediction System and the MetabolitePredict software, and a list of reported metabolites and TPs from the literature [5,15,16,40,41,51,53]. Samples were screened using specific criteria [52] and an in-house retention time prediction model [37]. Finally, the confidence levels for the identification of a compound proposed by Schymanski et al. (2014), were used [39].

### 4.6. Retrospective Screening of the Compounds in Wastewater

Retrospective analysis was done with the aim to assess the presence of PMMA, DHM, and their TPs in IWW and EWW. Specific criteria for the tentative identification or confirmation of these compounds were used, such as mass accuracy, isotopic fit, retention time window, and occurrence of qualifier ions (MS/MS spectra).

## Figures and Tables

**Table 1:** Transformation products identified for PMMA over seven-day incubation in the biotic reactor.

Compound	Retention Time (min.)	Measured *m/z* [M + H]^+^	Chemical Formula	Mass Error (Δm, ppm)	Diagnostic Product Ions (*m/z*)	Id. Level *
PMMA	4.38	180.1376	[C_11_H_18_NO]^+^	−3.89	149.0953, 121.0650, 109.0661, 91.0544	1
TP-166	3.01	166.1215	[C_10_H_16_NO]^+^	−6.62	135.0797, 107.0491	2a
TP-196a	3.03	196.1317	[C_11_H_17_NO_2_]^+^	−7.65	MS/MS spectra not obtained	4
TP-196b	3.53	196.1324	[C_11_H_17_NO_2_]^+^	−4.08	MS/MS spectra not obtained	4
TP-196c	3.93	196.1323	[C_11_H_17_NO_2_]^+^	−4.59	178.1216, 163.0981, 148.0736, 121.0654	3

* Identification level according to Schymanski et al. (2014) [39].

**Table 2:** Transformation products identified for DHM over seven-day incubation in the biotic reactor.

Compound	Retention Time (min.)	Measured *m/z* [M + H]^+^	Chemical Formula	Mass Error (Δm, ppm)	Diagnostic Product Ions (*m/z*)	Id. Level *
DHM	4.64	180.1382	[C_11_H_18_NO]^+^	−0.56	162.1286, 147.1047, 131.0861	1
TP-210	1.82	210.1130	[C_11_H_16_NO_3_]^+^	2.38	192.1011, 177.0722, 161.0577	3
TP-178 (mephedrone)	4.64	178.1212	[C_11_H_16_NO]^+^	−7.86	162.1269, 145.0883, 132.0826	1
TP-166	4.49	166.1228	[C_10_H_16_NO]^+^	1.20	148.112, 131.0848, 116.063	2b
TP-164	6.50	164.1071	[C_10_H_14_NO]^+^	0.61	146.0968, 131.0727, 120.0798	2b
TP-192a	5.10	192.1024	[C_11_H_14_NO_2_]^+^	2.60	148.1112, 135.0432, 119.049	3
TP-192b	6.15	192.1022	[C_11_H_14_NO_2_]^+^	1.56	148.1113, 135.0435, 119.0482	3

* Identification level according to Schymanski et al. (2014) [39].

## Data Availability

The data presented in this study are openly available in http://doi.org/10.3390/metabo11020066.

## References

[1] Peacock A., Bruno R., Gisev N., Degenhardt L., Hall W., Sedefov R., White J., Thomas K.V., Farrell M., Griffiths P. (2019). New psychoactive substances: Challenges for drug surveillance, control, and public health responses. Lancet.

[2] EMCDDA (2020). European Monitoring Centre for Drugs and Drug Addiction. European Drug Report—Trends and Developments.

[3] EMCDDA (2003). European Monitoring Centre for Drugs and Drug Addiction. Report on the Risk Assessment of PMMA in the Framework of the Joint Action on New Synthetic Drugs.

[4] Vevelstad M., Øiestad E.L., Nerem E., Arnestad M., Bogen I.L. (2017). Studies on Para-Methoxymethamphetamine (PMMA) Metabolite Pattern and Influence of CYP2D6 Genetics in Human Liver Microsomes and Authentic Samples from Fatal PMMA Intoxications. Drug Metab. Dispos..

[5] Kinyua J., Negreira N., McCall A.-K., Boogaerts T., Ort C., Covaci A., van Nuijs A.L.N. (2018). Investigating in-sewer transformation products formed from synthetic cathinones and phenethylamines using liquid chromatography coupled to quadrupole time-of-flight mass spectrometry. Sci. Total Environ..

[6] Mead J., Parrott A. (2020). Mephedrone and MDMA: A comparative review. Brain Res..

[7] Vevelstad M., Øiestad E.L., Middelkoop G., Hasvold I., Lilleng P., Delaveris G.J.M., Eggen T., Mørland J., Arnestad M. (2012). The PMMA epidemic in Norway: Comparison of fatal and non-fatal intoxications. Forensic Sci. Int..

[8] Lin D.-L., Liu H.-C., Yin H.-L. (2007). Recent Paramethoxymethamphetamine (PMMA) Deaths in Taiwan. J. Anal. Toxicol..

[9] Becker J., Neis P., Röhrich J., Zörntlein S. (2003). A fatal paramethoxymethamphetamine intoxication. Leg. Med..

[10] Johansen S.S., Carsten Hansen A., Müller I.B., Lundemose J.B., Franzmann M.-B. (2003). Three Fatal Cases of PMA and PMMA Poisoning in Denmark. J. Anal. Toxicol..

[11] Lurie Y., Gopher A., Lavon O., Almog S., Sulimani L., Bentur Y. (2012). Severe paramethoxymethamphetamine (PMMA) and paramethoxyamphetamine (PMA) outbreak in Israel. Clin. Toxicol..

[12] Nicol J.J.E., Yarema M.C., Jones G.R., Martz W., Purssell R.A., MacDonald J.C., Wishart I., Durigon M., Tzemis D., Buxton J.A. (2015). Deaths from exposure to paramethoxymethamphetamine in Alberta and British Columbia, Canada: A case series. CMAJ Open.

[13] Tang M.H., Ching C., Tse M., Ng C., Lee C., Chong Y., Wong W., Mak T.W.L. (2015). Surveillance of emerging drugs of abuse in Hong Kong: Validation of an analytical tools. Hong Kong Med. J..

[14] Pedersen A.J., Dalsgaard P.W., Rode A.J., Rasmussen B.S., Müller I.B., Johansen S.S., Linnet K. (2013). Screening for illicit and medicinal drugs in whole blood using fully automated SPE and ultra-high-performance liquid chromatography with TOF-MS with data-independent acquisition. J. Sep. Sci..

[15] Pedersen A.J., Reitzel L.A., Johansen S.S., Linnet K. (2013). In vitro metabolism studies on mephedrone and analysis of forensic cases. Drug Test. Anal..

[16] Meyer M.R., Wilhelm J., Peters F.T., Maurer H.H. (2010). Beta-keto amphetamines: Studies on the metabolism of the designer drug mephedrone and toxicological detection of mephedrone, butylone, and methylone in urine using gas chromatography–mass spectrometry. Anal. Bioanal. Chem..

[17] Kinyua J., Negreira N., Miserez B., Causanilles A., Emke E., Gremeaux L., de Voogt P., Ramsey J., Covaci A., van Nuijs A.L.N. (2016). Qualitative screening of new psychoactive substances in pooled urine samples from Belgium and United Kingdom. Sci. Total Environ..

[18] Lee H.H., Chen S.C., Lee J.F., Lin H.Y., Chen B.H. (2018). Simultaneous drug identification in urine of sexual assault victims by using liquid chromatography tandem mass spectrometry. Forensic Sci. Int..

[19] Bijlsma L., Celma A., López F.J., Hernández F. (2019). Monitoring new psychoactive substances use through wastewater analysis: Current situation, challenges and limitations. Curr. Opin. Environ. Sci. Health.

[20] González-Mariño I., Baz-Lomba J.A., Alygizakis N.A., Andrés-Costa M.J., Bade R., Bannwarth A., Barron L.P., Been F., Benaglia L., Berset J. (2020). Spatio-temporal assessment of illicit drug use at large scale: Evidence from 7 years of international wastewater monitoring. Addiction.

[21] O’Rourke C.E., Subedi B. (2020). Occurrence and Mass Loading of Synthetic Opioids, Synthetic Cathinones, and Synthetic Cannabinoids in Wastewater Treatment Plants in Four U.S. Communities. Environ. Sci. Technol..

[22] Bade R., White J.M., Nguyen L., Tscharke B.J., Mueller J.F., O’Brien J.W., Thomas K.V., Gerber C. (2020). Determining changes in new psychoactive substance use in Australia by wastewater analysis. Sci. Total Environ..

[23] Diamanti K., Aalizadeh R., Alygizakis N., Galani A., Mardal M., Thomaidis N.S. (2019). Wide-scope target and suspect screening methodologies to investigate the occurrence of new psychoactive substances in influent wastewater from Athens. Sci. Total Environ..

[24] Sulej-Suchomska A.M., Klupczynska A., Dereziński P., Matysiak J., Przybyłowski P., Kokot Z.J. (2020). Urban wastewater analysis as an effective tool for monitoring illegal drugs, including new psychoactive substances, in the Eastern European region. Sci. Rep..

[25] Bade R., Bijlsma L., Sancho J.V., Baz-Lomba J.A., Castiglioni S., Castrignanò E., Causanilles A., Gracia-Lor E., Kasprzyk-Hordern B., Kinyua J. (2017). Liquid chromatography-tandem mass spectrometry determination of synthetic cathinones and phenethylamines in influent wastewater of eight European cities. Chemosphere.

[26] Kinyua J., Covaci A., Maho W., McCall A.-K., Neels H., van Nuijs A.L.N. (2015). Sewage-based epidemiology in monitoring the use of new psychoactive substances: Validation and application of an analytical method using LC-MS/MS. Drug Test. Anal..

[27] Gracia-Lor E., Castiglioni S., Bade R., Been F., Castrignanò E., Covaci A., González-Mariño I., Hapeshi E., Kasprzyk-Hordern B., Kinyua J. (2017). Measuring biomarkers in wastewater as a new source of epidemiological information: Current state and future perspectives. Environ. Int..

[28] McCall A.-K., Bade R., Kinyua J., Lai F.Y., Thai P.K., Covaci A., Bijlsma L., van Nuijs A.L.N., Ort C. (2016). Critical review on the stability of illicit drugs in sewers and wastewater samples. Water Res..

[29] Gracia-Lor E., Sancho J.V., Serrano R., Hernández F. (2012). Occurrence and removal of pharmaceuticals in wastewater treatment plants at the Spanish Mediterranean area of Valencia. Chemosphere.

[30] Rousis N.I., Bade R., Bijlsma L., Zuccato E., Sancho J.V., Hernandez F., Castiglioni S. (2017). Monitoring a large number of pesticides and transformation products in water samples from Spain and Italy. Environ. Res..

[31] Gallé T., Koehler C., Plattes M., Pittois D., Bayerle M., Carafa R., Christen A., Hansen J. (2019). Large-scale determination of micropollutant elimination from municipal wastewater by passive sampling gives new insights in governing parameters and degradation patterns. Water Res..

[32] Kramer R.D., Filippe T.C., Prado M.R., de Azevedo J.C.R. (2018). The influence of solid-liquid coefficient in the fate of pharmaceuticals and personal care products in aerobic wastewater treatment. Environ. Sci. Pollut. Res..

[33] Mardal M., Meyer M.R. (2014). Studies on the microbial biotransformation of the novel psychoactive substance methylenedioxypyrovalerone (MDPV) in wastewater by means of liquid chromatography-high resolution mass spectrometry/mass spectrometry. Sci. Total Environ..

[34] Wick A., Wagner M., Ternes T.A. (2011). Elucidation of the Transformation Pathway of the Opium Alkaloid Codeine in Biological Wastewater Treatment. Environ. Sci. Technol..

[35] Davidsen A., Mardal M., Linnet K., Dalsgaard P.W. (2020). How to perform spectrum-based LC-HR-MS screening for more than 1,000 NPS with HighResNPS consensus fragment ions. PLoS ONE.

[36] Gago-Ferrero P., Schymanski E.L., Bletsou A.A., Aalizadeh R., Hollender J., Thomaidis N.S. (2015). Extended Suspect and Non-Target Strategies to Characterize Emerging Polar Organic Contaminants in Raw Wastewater with LC-HRMS/MS. Environ. Sci. Technol..

[37] Aalizadeh R., Nika M.-C., Thomaidis N.S. (2019). Development and application of retention time prediction models in the suspect and non-target screening of emerging contaminants. J. Hazard. Mater..

[38] Bade R., Abbate V., Abdelaziz A., Nguyen L., Trobbiani S., Stockham P., Elliott S., White J.M., Gerber C. (2020). The complexities associated with new psychoactive substances in influent wastewater: The case of 4-ethylmethcathinone. Drug Test. Anal..

[39] Schymanski E.L., Jeon J., Gulde R., Fenner K., Ruff M., Singer H.P., Hollender J. (2014). Identifying Small Molecules via High Resolution Mass Spectrometry: Communicating Confidence. Environ. Sci. Technol..

[40] Lai F.Y., Erratico C., Kinyua J., Mueller J.F., Covaci A., van Nuijs A.L.N. (2015). Liquid chromatography-quadrupole time-of-flight mass spectrometry for screening in vitro drug metabolites in humans: Investigation on seven phenethylamine-based designer drugs. J. Pharm. Biomed. Anal..

[41] Czerwinska J., Jang M., Costa C., Parkin M.C., George C., Kicman A.T., Bailey M.J., Dargan P.I., Abbate V. (2020). Detection of mephedrone and its metabolites in fingerprints from a controlled human administration study by liquid chromatography-tandem mass spectrometry and paper spray-mass spectrometry. Analyst.

[42] Gago-Ferrero P., Bletsou A.A., Damalas D.E., Aalizadeh R., Alygizakis N.A., Singer H.P., Hollender J., Thomaidis N.S. (2020). Wide-scope target screening of >2000 emerging contaminants in wastewater samples with UPLC-Q-ToF-HRMS/MS and smart evaluation of its performance through the validation of 195 selected representative analytes. J. Hazard. Mater..

[43] Huntscha S., Hofstetter T.B., Schymanski E.L., Spahr S., Hollender J. (2014). Biotransformation of Benzotriazoles: Insights from Transformation Product Identification and Compound-Specific Isotope Analysis. Environ. Sci. Technol..

[44] Baselt R.C. (2014). Disposition of Toxic Drugs and Chemicals in Man.

[45] Ort C., van Nuijs A.L.N., Berset J.-D., Bijlsma L., Castiglioni S., Covaci A., de Voogt P., Emke E., Fatta-Kassinos D., Griffiths P. (2014). Spatial differences and temporal changes in illicit drug use in Europe quantified by wastewater analysis. Addiction.

[46] Castrignanò E., Yang Z., Bade R., Baz-Lomba J.A., Castiglioni S., Causanilles A., Covaci A., Gracia-Lor E., Hernandez F., Kinyua J. (2018). Enantiomeric profiling of chiral illicit drugs in a pan-European study. Water Res..

[47] González-Mariño I., Gracia-Lor E., Rousis N.I., Castrignanò E., Thomas K.V., Quintana J.B., Kasprzyk-Hordern B., Zuccato E., Castiglioni S. (2016). Wastewater-Based Epidemiology To Monitor Synthetic Cathinones Use in Different European Countries. Environ. Sci. Technol..

[48] Fallati L., Castiglioni S., Galli P., Riva F., Gracia-Lor E., González-Mariño I., Rousis N.I., Shifah M., Messa M.C., Strepparava M.G. (2020). Use of legal and illegal substances in Malé (Republic of Maldives) assessed by wastewater analysis. Sci. Total Environ..

[49] Gao J., Banks A., Li J., Jiang G., Lai F.Y., Mueller J.F., Thai P.K. (2017). Evaluation of in-sewer transformation of selected illicit drugs and pharmaceutical biomarkers. Sci. Total Environ..

[50] Linhart I., Himl M., Židková M., Balíková M., Lhotková E., Páleníček T. (2016). Metabolic profile of mephedrone: Identification of nor-mephedrone conjugates with dicarboxylic acids as a new type of xenobiotic phase II metabolites. Toxicol. Lett..

[51] Czerwinska J., Parkin M.C., George C., Kicman A.T., Dargan P.I., Abbate V. (2020). Pharmacokinetics of Mephedrone and Its Metabolites in Whole Blood and Plasma after Controlled Intranasal Administration to Healthy Human Volunteers. J. Anal. Toxicol..

[52] Beretsou V.G., Psoma A.K., Gago-Ferrero P., Aalizadeh R., Fenner K., Thomaidis N.S. (2016). Identification of biotransformation products of citalopram formed in activated sludge. Water Res..

[53] Staack R., Fehn J., Maurer H. (2003). New designer drug p-methoxymethamphetamine: Studies on its metabolism and toxicological detection in urine using gas chromatography–mass spectrometry1. J. Chromatogr. B.

